# The clinical value of mNGS of bronchoalveolar lavage fluid versus traditional microbiological tests for pathogen identification and prognosis of severe pneumonia (NT-BALF):study protocol for a prospective multi-center randomized clinical trial

**DOI:** 10.1186/s13063-024-08112-x

**Published:** 2024-04-22

**Authors:** Xiao Song, Hui Jiang, Liang Zong, Di Shi, Huadong Zhu

**Affiliations:** Emergency Department, The State Key Laboratory for Complex, Severe and Rare Diseases, Peking Union Medical College Hospital, Chinese Academy of Medical Science and Peking Union Medical College, Beijing, China

**Keywords:** Severe pneumonia, mNGS, Traditional microbiological tests, Bronchoalveolar lavage fluid, Cost-effectiveness

## Abstract

**Background:**

Early, rapid, and accurate pathogen diagnosis can help clinicians select targeted treatment options, thus improving prognosis and reducing mortality rates of severe pneumonia. Metagenomic next-generation sequencing (mNGS) has a higher sensitivity and broader pathogen spectrum than traditional microbiological tests. However, the effects of mNGS-based antimicrobial treatment procedures on clinical outcomes and cost-effectiveness in patients with severe pneumonia have not been evaluated.

**Methods:**

This is a regional, multi-center, open, prospective, randomized controlled trial to evaluate that whether the combination of mNGS and traditional testing methods could decrease 28-day call-cause mortality with moderate cost-effectiveness. A total of 192 patients with severe pneumonia will be recruited from four large tertiary hospitals in China. Bronchoalveolar lavage fluid will be obtained in all patients and randomly assigned to the study group (mNGS combined with traditional microbiological tests) or the control group (traditional microbiological tests only) in a 1:1 ratio. Individualized antimicrobial treatment and strategy will be selected according to the analysis results. The primary outcome is 28-day all-cause mortality. The secondary outcomes are ICU and hospital length of stay (LOS), ventilator-free days and ICU-free days, consistency between mNGS and traditional microbiological tests, detective rate of mNGS and traditional microbiological tests, turn-out time, time from group allocation to start of treatment, duration of vasopressor support, types and duration of anti-infective regimens, source of drug-resistant bacteria or fungi, and ICU cost.

**Discussion:**

The clinical benefits of mNGS are potentially significant, but its limitations should also be considered.

**Trial registration:**

ChineseClinicalTrialRegistry.org, ChiCTR2300076853. Registered on 22 October 2023.

**Supplementary Information:**

The online version contains supplementary material available at 10.1186/s13063-024-08112-x.

## Introduction

### Background

Severe pneumonia caused by various pathogenic microorganisms is a leading cause of hospitalization and death worldwide [1]. The inappropriate application of antibiotics for pneumonia at early stages is the major reason for the increased morbidity and mortality rates [2]. If treatment is delayed, severe pneumonia can progress to multiple organ dysfunction and even death. Despite significant advances in etiological research and antimicrobial treatment, severe pneumonia remains the leading cause of death from infectious diseases worldwide [3]. In addition to the necessary mechanical ventilation, early, rapid, and accurate pathogen diagnosis can help clinicians select targeted treatment options, thus improving prognosis and reducing mortality rates [4].

Traditional microbiological tests include smear, culture, serology, and limited molecular panels. These methods are unable to identify the full range of potential pathogens (e.g., fastidious or nonculturable organisms or uncommon organisms not included in current panels). In addition, in many clinical laboratories, culture is very time consuming, requiring a 3–5-day wait time to get an accurate report. Serological testing and PCR require a priori assumption about the presence of a particular virus and require the laboratory to have the appropriate commercial kit. These conditions often lead to missed detection of certain pathogens [5]. Previous studies have shown that metagenomic next-generation sequencing (mNGS) has a higher sensitivity and broader pathogen spectrum than traditional microbiological tests. Recently, the possibility of using mNGS to identify non-sterile site specimens in the respiratory tract has been discussed. mNGS is assumed to optimize antibiotic treatment in critically ill patients with infectious diseases. While helpful, little is known about how to interpret the application of mNGS results in lower respiratory tract infections (LRTIs), which leads to conflicting opinions, as mNGS does not elucidate whether the organism is viable or a pathogen. Incorrect interpretation or false positive results perhaps escalate antimicrobial regimen [6]. This may result in inappropriate treatment, which may prolong or aggravate the status of disease. In particular, prospective clinical trial and economic data showing the cost-effectiveness of these relatively expensive tests in improving patient outcomes are needed to justify their use.

#### Objectives

Recently, the possibility of using mNGS to identify non-sterile site specimens in the respiratory tract has been discussed, though there are still conflicting opinions. The purpose of this study is to evaluate the effectiveness and consistency of bronchoalveolar lavage fluid (BALF) mNGS in diagnosing and treating pulmonary infections in comparison to conventional testing methods. Furthermore, the aim is to demonstrate whether the combination of mNGS and traditional testing methods could decrease 28-day all-cause mortality with moderate cost-effectiveness.

## Method

### Study design

**Fig. 1:**
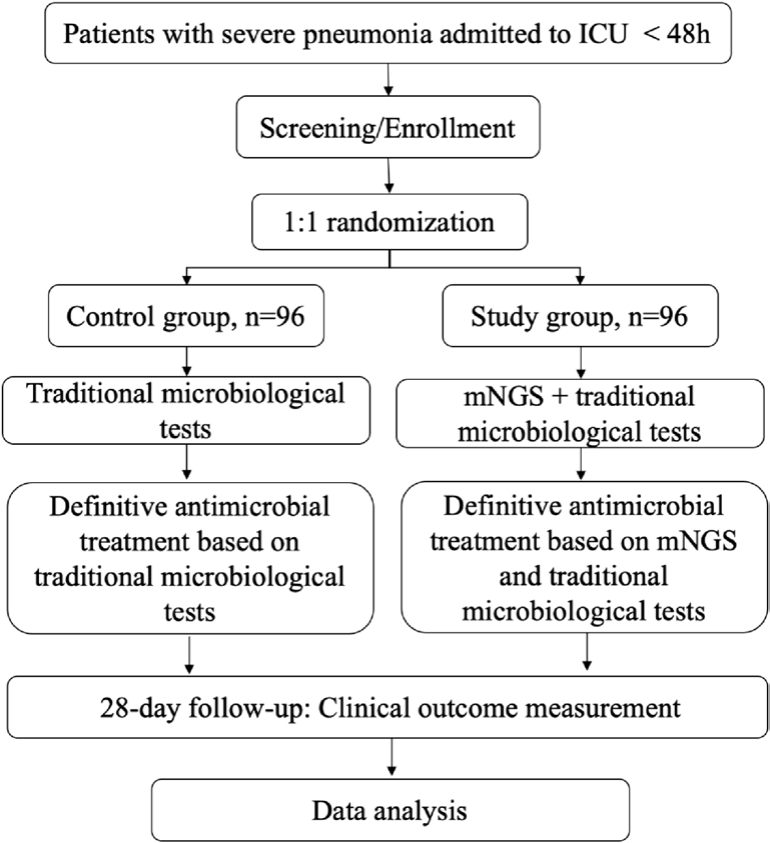
Flowchart of the study protocol

**Fig. 2:**
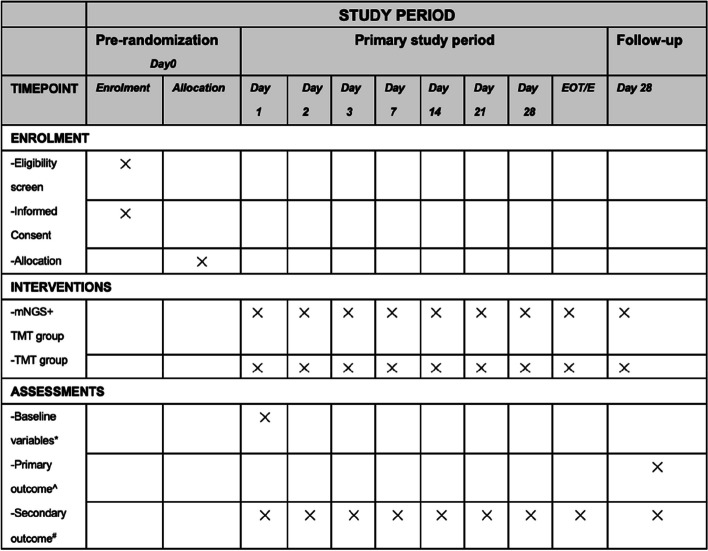
The schedule of enrolment, intervention and assessments. TMT, traditional microbiological tests; EOT, end of therapy; E, discharge from the ICU. “*” symbol indicates baseline variables: age, gender, BMI (body mass index), history of smoking, comorbidity, etc. “^” symbol indicates primary outcome: 28-day all-cause mortality. “^#^” symbol indicates secondary outcome: (1) ICU and hospital length of stay (LOS), (2) ventilator-free days and ICU-free days, (3) consistency between mNGS and traditional microbiological tests, (4) detective rate of mNGS and traditional microbiological tests, (5) turn-out time, (6) types and duration of anti-infective regimens, (7) source of drug-resistant bacteria or fungi, and (8) ICU cost

This study is a regional, multi-center, open, prospective, randomized controlled trial that will include patients with severe pneumonia, from four large tertiary hospitals in North China. A total of 192 participants will be enrolled and randomly assigned in a 1:1 ratio to either the study group (mNGS combined with traditional microbiological tests group) or the control group (traditional microbiological tests group) using the central randomization system. Patients will be provided anti-infective treatment based on clinical experience according to mNGS + traditional tests or traditional tests only for two groups. The NT-BALF trial will be conducted according to the principles of the Declaration of Helsinki [7]. The Institutional Review Board of Peking Union Medical College Hospital, Chinese Academy of Medical Sciences & Peking Union Medical College in Beijing, China, approved the trial protocol (version 4.0, reference number I-23PJ1316, date 15 August 2023). The flowchart of the protocol is shown in Fig. 1. The schedule of enrolment, intervention, and assessments following the Standard Protocol Items: Recommendation for Interventional Trials (SPIRIT) guidelines (Additional file 1) is presented in Fig. 2.

### Inclusion criteria and exclusion criteria

Inclusion criteria are (1) patient aged ≥ 18 years, (2) diagnosis of severe pneumonia and admitted to an ICU, and (3) time from severe pneumonia diagnosis to ICU admission < 48 h. Severe pneumonia was defined in patients with either one major criterion or at least three minor criteria of the Infectious Diseases Society of America (IDSA)/American Thoracic Society (ATS) criteria [8]. Major criteria are as follows: (1) septic shock with need for vasopressors and (2) respiratory failure requiring mechanical ventilation. Minor criteria are as follows: (1) respiratory rate > 30 breaths/min, (2) PaO_2_/FIO_2_ ratio < 250, (3) multi-lobar infiltrates, (4) confusion/disorientation, (5) uremia (blood urea nitrogen level > 20 mg/dl), (6) leukopenia (white blood cell count < 4000 cells/ml), (7) thrombocytopenia (platelet count < 100,000/ml), (8) hypothermia (core temperature < 36 °C), (9) hypotension requiring aggressive fluid resuscitation. Informed consent will be obtained from all the patients or their guardians before enrollment. Participants will be excluded if they are (1) aged < 18 years, (2) pregnant or lactating, (3) are expected to die within 24 h, (4) diagnosed with HIV, or (5) are receiving palliative therapy or supportive treatment only.

### Participant recruitment

Participants will be recruited from four medical centers in tertiary hospitals in North China including Peking Union Medical College Hospital, Beijing Chao-yang Hospital, Tianjin Medical University General Hospital, and The Second Hospital of Hebei Medical University. There is a research coordinator at each hospital to promote and coordinate the trial. Patients will be informed of the purpose and procedures involved and the potential risks and benefits of the study. The participants’ written consent will be obtained. Participants will be allowed to withdraw from the trial at any time without consequence.

Each branch center will post printed recruitment posters inside and outside the hospital. Patients with severe pneumonia will be invited to participate in this study once after diagnosis. At the screening visit, a trained research staff (physicians) will confirm the eligibility by the symptom, laboratory result, and chest X-ray or CT image and check every inclusion and exclusion criterion. The recruitment time is 36 months, from November 2023 to October 2026.

### Randomization and blinding

Stratified block randomization will be performed centrally in a 1:1 ratio using the SAS software (SAS Institute, Inc., Cary, NC, USA). Within each stratum, a random block size of 4 will be used. Considering the nature of antibiotic intervention, this study is open to the clinicians, patients, and investigator; only the data analyst and follow-up evaluator will be blinded.

### Intervention

For both groups, bronchoalveolar lavage fluid will be harvested within 24 h of participants entering the ICU. Traditional microbiological tests will be collected as soon as possible after admission and will be performed in local in-house laboratories. Traditional microbiological tests, including culture, antigen detection, multiplex PCR, and direct microscopic examination of specimens. The results of traditional microbiological tests will be interpreted according to standard procedures.

BALF specimens will be divided into aliquots and used for both mNGS tests and traditional microbiological tests for study group. BALF mNGS tests will be transferred to the same professional genomic laboratory independently by cold chain transportation. The genomic laboratory will perform nucleic acid extraction, library construction, amplification and sequencing, bioinformatic analysis, and data interpretation according to previous clinical practice.

In the study group, anti-infective treatment regimen is based on both BALF mNGS and traditional microbiological tests, while in the control group, it is based on the results of traditional microbiological tests. The results of mNGS and necessary clinical data will be judged by a group of senior clinicians, who reached a unified opinion.

### Data measurement

Clinical data will be collected locally on an electronic case report form (CRF). The research coordinator at each hospital will provide training in study procedures to improve adherence to the protocol. Furthermore, they will check CRFs and contact responsible staff members to ensure data quality.

### Outcomes

The primary outcome is 28-day all-cause mortality. The secondary outcomes are (1) ICU and hospital length of stay (LOS), (2) ventilator-free days and ICU-free days, (3) consistency between mNGS and traditional microbiological tests, (4) detective rate of mNGS and traditional microbiological tests, (5) turn-out time, (6) types and duration of anti-infective regimens, (7) time from group allocation to start of treatment, (8) duration of vasopressor support, (9) source of drug-resistant bacteria or fungi, and (10) ICU cost.

### Adverse events and serious adverse events

Possible adverse events of bronchoalveolar lavage include anesthesia accidents, bronchospasm, pneumothorax or mediastinal emphysema, exacerbation of hypoxia, and respiratory or cardiac arrest. Record whether each subject undergoing bronchoalveolar lavage experiences adverse events and what specific adverse events occur. Serious adverse events are defined as respiratory or cardiac arrest or any adverse event that leads to the termination of bronchoalveolar lavage procedure.

### Sample size

In this study, a randomized controlled trial design is used to evaluate the treatment effect of mNGS and traditional microbiological tests in patients with severe pneumonia. According to a previous report [9], we hypothesized the 28-day all-cause mortality of study group and control group to be 15% and 33%, respectively. We assumed that *α* = 0.05 (two-sided test) and *β* = 0.20. Eighty-five patients should be recruited in each group. Considering an attrition rate of 10%, the eligible participants in each group should be 96. Therefore, we determined that the sample size should be 95 in each group (*n* = 192 in total).

### Statistical analysis

Data will be stored electronically and analyzed using the R software. A 2-sided *P* < 0.05 will be used as the statistical significance threshold. For continuous variables with nonparametric distribution, the data will be expressed as medians and IQRs, and the Mann-Whitney test will be used to compare outcomes. For variables with normal distribution, the data will be expressed as means and SDs, and the *t* test will be used to compare outcomes. The between-group differences will be reported as mean differences with 95% CIs. Two-sided significance tests will be applied to all analyses.

We will compare dichotomous data using the 2-tailed *χ*^2^ test when the number of variables is more than 5 and using the Fisher exact test when the number of variables is equal to or less than 5. We will calculate relative risks and 95% CIs using the 2 × 2 table method with log-linear regression and a normal approximation for the SE.

The survival analysis of the primary outcome will be tested based on the intention-to-treat principle using the Kaplan–Meier method, and the existence of statistical significance between the survival curves will be compared using a log-rank test. Subgroup analyses will be performed to identify potential modifiers of the intervention effect, including gender, age, smoking history, comorbidities, inflammatory indicators, antibiotics use, the feature of chest X-ray or computed tomography (CT) image, the way of oxygen therapy, and the severity scores.

### Quality control

A data monitoring committee, comprising physicians, an intensivist, a microbiologist, a statistician, and quality management personnel, will be established. Before the study begin, the principal investigators will accept good clinical practice (GCP) training, and all researchers will accept training including study protocol, informed consent, case report form, standard operating procedures of participants’ data collections, and collection and preservation methods of biological samples to ensure the trail quality. They not only determine the methods of clinical trials but also solve major problems encountered in practical applications. Participants will accept bronchoscopy by trained respiratory physician to acquire lower respiratory samples within 24 h. Biological samples for mNGS testing will be transferred to the same professional genomic laboratory (Vision Medicals Center for Infectious Diseases) independently by cold chain transportation. During the study, samples for traditional microbiological testing will be conducted by trained specialists of local in-house laboratories with standard procedure.

### Plans for communicating important protocol amendments to relevant parties

An “important protocol modifications” is defined as an amendment to the protocol or any other supporting documentation that is likely to affect to a significant degree the safety or physical integrity of the subjects, the scientific value of the trial, and the conduct or management of the trial. All substantial amendments will be notified to research coordinators, the data monitoring committee, and the competent authority. Non-substantial amendments will be recorded and filed. In case amendments concern or affect participants in any way, they will be informed about the changes. If needed, additional consent will be requested and registered. Moreover, online trial registries will be updated accordingly.

## Discussion

Over the years, traditional microbiological methods for pulmonary infective diseases include a variety of culture-based approaches, visualization by microscopy, antigen detection, serological methods, and molecular detection. Recently, metagenomic NGS has become a well-established method in clinical microbiology laboratories to aid in the diagnosis of infectious diseases. Next-generation sequencing was originally used for applications such as oncology and inherited disease diagnosis. Targeted traditional microbiological methods detect a defined spectrum of organisms. By contrast, untargeted mNGS offers the unique advantage of broadly identifying organisms, particularly those are fastidious or non-culturable or rapid diagnostics unavailable [6, 10]. Furthermore, mNGS has demonstrated improved detection of mixed pulmonary infections in relation to traditional microbiological tests [11].

The clinical benefits of mNGS are potentially significant, but its limitations should also be considered. A significant potential limitation of mNGS assays for infectious disease diagnostics is the possibility of false-positive results due to contamination. Contamination of respiratory specimens by normal microbiota during the process of collection is highly likely, as the respiratory tract is not a sterile system [12]. Thus, quality control for mNGS assays is critical to ensure accurate performance. In our study, the establishment and monitoring of robust mNGS quality control metrics have been successfully adapted to the clinical microbiology laboratory setting despite of complex multistep tests. Moreover, considered as sterile respiratory specimen, BALF will be collected in this study.

Another potential limitation of mNGS assays for infectious disease diagnostics is that mNGS provides valuable information regarding the presence of genetic material but does not elucidate whether the organism is viable or a pathogen [12]. The interpretation and reporting of mNGS data from a respiratory sample are complex. Interpreting organisms without clear clinical presentation, patient history and risk factors, and results from traditional microbiological tests could lead to misdiagnosis and inappropriate treatment, negatively impacting patient care [13]. In this study, we will evaluate clinical effectiveness and risks of mNGS based on traditional microbiological tests, namely primary and secondary outcomes.

Studies regarding the cost-effectiveness of mNGS assays for pathogen detection are lacking. Relative to most traditional microbiological tests, mNGS testing is expensive. The incremental costs of testing are minimal, compared to the costs of intensive care unit stays or myriad diagnostic tests, not to mention use of even more expensive and advanced anti-infective drugs that are ordered for complex cases. Our study will evaluate the cost-effectiveness data of mNGS testing in severe pneumonia.

## Trial status

Recruiting will be started in November 2023. The current protocol is version 4 (9 August 2023). Patient recruitment is estimated to be completed around October 2026.

### Supplementary Information

**Additional file 1.** SPIRIT checklist.

**Additional file 2.** Informed consent.

## Data Availability

The datasets generated and/or analyzed during the current study are available from the principal investigator (Xiao Song and Di Shi) on reasonable request.

## Abbreviations

mNGS: Metagenomic next-generation sequencing
LOS: Length of stay
LRTIs: Lower respiratory tract infections
BALF: Bronchoalveolar lavage fluid
TMT: Traditional microbiological tests
EOT: End of therapy
IDSA: Infectious Diseases Society of America
ATS: American Thoracic Society
CRF: Case report form
CT: Computed tomography
GCP: Good clinical practice

## References

[1] Thomas CP, Ryan M, Chapman JD, Stason WB, Tompkins CP, Suaya JA, Polsky D, Mannino DM, Shepard DS (2012). Incidence and cost of pneumonia in medicare beneficiaries. Chest.

[2] Postma DF, van Werkhoven CH, van Elden LJ, Thijsen SF, Hoepelman AI, Kluytmans JA, Boersma WG, Compaijen CJ, van der Wall E, Prins JM (2015). Antibiotic treatment strategies for community-acquired pneumonia in adults. N Engl J Med.

[3] De Pascale G, Bello G, Antonelli M (2011). Steroids in severe pneumonia: a literature review. Minerva anestesiologica.

[4] Cillóniz C, Torres A, Niederman MS (2021). Management of pneumonia in critically ill patients. BMJ.

[5] Li G, Huang J, Li Y, Feng J (2020). The value of combined radial endobronchial ultrasound-guided transbronchial lung biopsy and metagenomic next-generation sequencing for peripheral pulmonary infectious lesions. Can Respir J.

[6] Chiu CY, Miller SA (2019). Clinical metagenomics. Nat Rev Genet.

[7] General Assembly of the World Medical A (2014). World Medical Association Declaration of Helsinki: ethical principles for medical research involving human subjects. J Am Coll Dent.

[8] Metlay JP, Waterer GW, Long AC, Anzueto A, Brozek J, Crothers K, Cooley LA, Dean NC, Fine MJ, Flanders SA (2019). Diagnosis and treatment of adults with community-acquired pneumonia. an official clinical practice guideline of the American Thoracic Society and Infectious Diseases Society of America. Am J Respir Crit Care Med.

[9] Lv M, Zhu C, Zhu C, Yao J, Xie L, Zhang C, Huang J, Du X, Feng G (2023). Clinical values of metagenomic next-generation sequencing in patients with severe pneumonia: a systematic review and meta-analysis. Front Cell Infect Microbiol.

[10] Miller S, Chiu C, Rodino KG, Miller MB (2020). Point-counterpoint:should we be performing metagenomic next-generation sequencing for infectious disease diagnosis in the clinical laboratory?. J Clin Microbiol.

[11] Qu J, Zhang J, Chen Y, Huang Y, Xie Y, Zhou M, Li Y, Shi D, Xu J, Wang Q (2022). Aetiology of severe community acquired pneumonia in adults identified by combined detection methods: a multi-centre prospective study in China. Emerg Microbes Infect.

[12] Maljkovic Berry I, Melendrez MC, Bishop-Lilly KA, Rutvisuttinunt W, Pollett S, Talundzic E, Morton L, Jarman RG (2020). Next generation sequencing and bioinformatics methodologies for infectious disease research and public health: approaches, applications, and considerations for development of laboratory capacity. J Infect Dis.

[13] Miller S, Chiu C (2021). The role of metagenomics and next-generation sequencing in infectious disease diagnosis. Clin Chem.

