# Supplementary material for: The clinical value of mNGS of bronchoalveolar lavage fluid versus traditional microbiological tests for pathogen identification and prognosis of severe pneumonia (NT-BALF):study protocol for a prospective multi-center randomized clinical trial
Source: Trials. 2024 Apr 22;25:276. doi: 10.1186/s13063-024-08112-x (PMC11036641; doi:10.1186/s13063-024-08112-x)
Supplement: Supplementary file 1 — Additional file 1. SPIRIT checklist. [file 13063_2024_8112_MOESM1_ESM.docx]

**Additional file 1：Spirit checklist**

Reporting checklist for protocol of a clinical trial.

Based on the SPIRIT guidelines.

**Instructions to authors**

Complete this checklist by entering the page numbers from your manuscript where readers will find each of the items listed below.

Your article may not currently address all the items on the checklist. Please modify your text to include the missing information. If you are certain that an item does not apply, please write "n/a" and provide a short explanation.

Upload your completed checklist as an extra file when you submit to a journal.

In your methods section, say that you used the SPIRITreporting guidelines, and cite them as:

Chan A-W, Tetzlaff JM, Gøtzsche PC, Altman DG, Mann H, Berlin J, Dickersin K, Hróbjartsson A, Schulz KF, Parulekar WR, Krleža-Jerić K, Laupacis A, Moher D. SPIRIT 2013 Explanation and Elaboration: Guidance for protocols of clinical trials. BMJ. 2013;346:e7586

|  | | Reporting Item | Page  Number |
| --- | --- | --- | --- |
| **Administrative** |  |  |  |
| **information** |  |  |  |
| Title | [#1](https://www.goodreports.org/reporting-checklists/spirit/info/#1) | Descriptive title identifying the study design, population, | 1 |
|  |  | interventions, and, if applicable, trial acronym |  |
| Trial registration | [#2a](https://www.goodreports.org/reporting-checklists/spirit/info/#2a) | Trial identifier and registry name. If not yet registered, | 1 |
|  |  | name of intended registry |  |
| Trial registration: data set | [#2b](https://www.goodreports.org/reporting-checklists/spirit/info/#2b) | All items from the World Health Organization Trial Registration Data Set | 1 |
| Protocol version | [#3](https://www.goodreports.org/reporting-checklists/spirit/info/#3) | Date and version identifier | 8 |
| Funding | [#4](https://www.goodreports.org/reporting-checklists/spirit/info/#4) | Sources and types of financial, material, and other support | 8 |

Roles and responsibilities: contributorship

[#5a](https://www.goodreports.org/reporting-checklists/spirit/info/#5a) Names, affiliations, and roles of protocol contributors 1,9

Roles and responsibilities: sponsor contact information

[#5b](https://www.goodreports.org/reporting-checklists/spirit/info/#5b) Name and contact information for the trial sponsor 8

Roles and responsibilities: sponsor and funder

[#5c](https://www.goodreports.org/reporting-checklists/spirit/info/#5c) Role of study sponsor and funders, if any, in study design; 8

collection, management, analysis, and interpretation of data; writing of the report; and the decision to submit the report for publication, including whether they will have ultimate authority over any of these activities

Roles and responsibilities: committees

[#5d](https://www.goodreports.org/reporting-checklists/spirit/info/#5d) Composition, roles, and responsibilities of the 9

coordinating centre, steering committee, endpoint adjudication committee, data management team, and other individuals or groups overseeing the trial, if applicable (see Item 21a for data monitoring committee)

# Introduction

Background and rationale

[#6a](https://www.goodreports.org/reporting-checklists/spirit/info/#6a) Description of research question and justification for undertaking the trial, including summary of relevant studies (published and unpublished) examining benefits and harms for each intervention

2

Background and rationale: choice of comparators

[#6b](https://www.goodreports.org/reporting-checklists/spirit/info/#6b) Explanation for choice of comparators 2

Objectives [#7](https://www.goodreports.org/reporting-checklists/spirit/info/#7) Specific objectives or hypotheses 2

Trial design [#8](https://www.goodreports.org/reporting-checklists/spirit/info/#8) Description of trial design including type of trial (eg, 2-3

parallel group, crossover, factorial, single group), allocation ratio, and framework (eg, superiority, equivalence, non-inferiority, exploratory)

# Methods: Participants, interventions, and

**outcomes**

Study setting [#9](https://www.goodreports.org/reporting-checklists/spirit/info/#9) Description of study settings (eg, community clinic,

academic hospital) and list of countries where data will be collected. Reference to where list of study sites can be obtained

2-3

Eligibility criteria [#10](https://www.goodreports.org/reporting-checklists/spirit/info/#10) Inclusion and exclusion criteria for participants. If

applicable, eligibility criteria for study centres and individuals who will perform the interventions (eg, surgeons, psychotherapists)

4-5

Interventions: description

|  | | administered | Fig. 1 |
| --- | --- | --- | --- |
| Interventions: | [#11b](https://www.goodreports.org/reporting-checklists/spirit/info/#11b) | Criteria for discontinuing or modifying allocated | 6 |
| modifications |  | interventions for a given trial participant (eg, drug dose |  |
|  |  | change in response to harms, participant request, or |  |
|  |  | improving / worsening disease) |  |
| Interventions: adherance | [#11c](https://www.goodreports.org/reporting-checklists/spirit/info/#11c) | Strategies to improve adherence to intervention protocols, and any procedures for monitoring adherence (eg, drug | 6-7 |
|  |  | tablet return; laboratory tests) |  |
| Interventions: | [#11d](https://www.goodreports.org/reporting-checklists/spirit/info/#11d) | Relevant concomitant care and interventions that are | 5 |
| concomitant care |  | permitted or prohibited during the trial |  |
| Outcomes | [#12](https://www.goodreports.org/reporting-checklists/spirit/info/#12) | Primary, secondary, and other outcomes, including the | 6 |
|  |  | specific measurement variable (eg, systolic blood |  |
|  |  | pressure), analysis metric (eg, change from baseline, final |  |
|  |  | value, time to event), method of aggregation (eg, median,  proportion), and time point for each outcome. Explanation |  |
|  |  | of the clinical relevance of chosen efficacy and harm |  |
|  |  | outcomes is strongly recommended |  |
| Participant timeline | [#13](https://www.goodreports.org/reporting-checklists/spirit/info/#13) | Time schedule of enrolment, interventions (including any | 4-5, |
|  |  | run-ins and washouts), assessments, and visits for | Fig.2 |
|  |  | participants. A schematic diagram is highly recommended (see Figure) |  |
| Sample size | [#14](https://www.goodreports.org/reporting-checklists/spirit/info/#14) | Estimated number of participants needed to achieve study objectives and how it was determined, including | 6 |

[#11a](https://www.goodreports.org/reporting-checklists/spirit/info/#11a) Interventions for each group with sufficient detail to allow replication, including how and when they will be

5

clinical and statistical assumptions supporting any sample size calculations

Recruitment [#15](https://www.goodreports.org/reporting-checklists/spirit/info/#15) Strategies for achieving adequate participant enrolment to 4-5

reach target sample size

# Methods: Assignment of interventions (for controlled trials)

Allocation: sequence generation

[#16a](https://www.goodreports.org/reporting-checklists/spirit/info/#16a) Method of generating the allocation sequence (eg, 5

computer-generated random numbers), and list of any factors for stratification. To reduce predictability of a random sequence, details of any planned restriction (eg, blocking) should be provided in a separate document that is unavailable to those who enrol participants or assign interventions

Allocation concealment mechanism

[#16b](https://www.goodreports.org/reporting-checklists/spirit/info/#16b) Mechanism of implementing the allocation sequence (eg, 5

central telephone; sequentially numbered, opaque, sealed envelopes), describing any steps to conceal the sequence until interventions are assigned

Allocation: implementation

[#16c](https://www.goodreports.org/reporting-checklists/spirit/info/#16c) Who will generate the allocation sequence, who will enrol 4-5

participants, and who will assign participants to interventions

Blinding (masking) [#17a](https://www.goodreports.org/reporting-checklists/spirit/info/#17a) Who will be blinded after assignment to interventions (eg, 5

trial participants, care providers, outcome assessors, data analysts), and how

Not applicable,

open labelled

Blinding (masking): emergency unblinding

[#17b](https://www.goodreports.org/reporting-checklists/spirit/info/#17b) If blinded, circumstances under which unblinding is

permissible, and procedure for revealing a participant’s allocated intervention during the trial

# Methods: Data collection, management, and analysis

Data collection plan [#18a](https://www.goodreports.org/reporting-checklists/spirit/info/#18a) Plans for assessment and collection of outcome,

baseline, and other trial data, including any related

4-5

|  | | processes to promote data quality (eg, duplicate  measurements, training of assessors) and a description of |  |
| --- | --- | --- | --- |
|  |  | study instruments (eg, questionnaires, laboratory tests) |  |
|  |  | along with their reliability and validity, if known. Reference  to where data collection forms can be found, if not in the |  |
|  |  | protocol |  |
| Data collection plan: retention | [#18b](https://www.goodreports.org/reporting-checklists/spirit/info/#18b) | Plans to promote participant retention and complete follow-up, including list of any outcome data to be | 4-5 |
|  |  | collected for participants who discontinue or deviate from |  |
|  |  | intervention protocols |  |
| Data management | [#19](https://www.goodreports.org/reporting-checklists/spirit/info/#19) | Plans for data entry, coding, security, and storage, including any related processes to promote data quality | 5 |
|  |  | (eg, double data entry; range checks for data values). |  |
|  |  | Reference to where details of data management |  |
|  |  | procedures can be found, if not in the protocol |  |
| Statistics: outcomes | [#20a](https://www.goodreports.org/reporting-checklists/spirit/info/#20a) | Statistical methods for analysing primary and secondary | 6 |
|  |  | outcomes. Reference to where other details of the |  |
|  |  | statistical analysis plan can be found, if not in the protocol |  |
| Statistics: additional analyses | [#20b](https://www.goodreports.org/reporting-checklists/spirit/info/#20b) | Methods for any additional analyses (eg, subgroup and adjusted analyses) | 6 |
| Statistics: analysis population and | [#20c](https://www.goodreports.org/reporting-checklists/spirit/info/#20c) | Definition of analysis population relating to protocol non- adherence (eg, as randomised analysis), and any |  |
| missing data |  | statistical methods to handle missing data (eg, multiple |  |
|  |  | imputation) |  |
| **Methods: Monitoring** |  |  |  |
| Data monitoring: | [#21a](https://www.goodreports.org/reporting-checklists/spirit/info/#21a) | Composition of data monitoring committee (DMC); | 6-7 |
| formal committee |  | summary of its role and reporting structure; statement of |  |
|  |  | whether it is independent from the sponsor and |  |
|  |  | competing interests; and reference to where further  details about its charter can be found, if not in the |  |
|  |  | protocol. Alternatively, an explanation of why a DMC is |  |
|  |  | not needed |  |
| Data monitoring: | [#21b](https://www.goodreports.org/reporting-checklists/spirit/info/#21b) | Description of any interim analyses and stopping | 5 |
| interim analysis |  | guidelines, including who will have access to these |  |

interim results and make the final decision to terminate the trial

Harms [#22](https://www.goodreports.org/reporting-checklists/spirit/info/#22) Plans for collecting, assessing, reporting, and managing 6-7

solicited and spontaneously reported adverse events and other unintended effects of trial interventions or trial conduct

Auditing [#23](https://www.goodreports.org/reporting-checklists/spirit/info/#23) Frequency and procedures for auditing trial conduct, if 5,9

any, and whether the process will be independent from investigators and the sponsor

# Ethics and dissemination

Research ethics approval

[#24](https://www.goodreports.org/reporting-checklists/spirit/info/#24) Plans for seeking research ethics committee / institutional review board (REC / IRB) approval

8

Protocol amendments

[#25](https://www.goodreports.org/reporting-checklists/spirit/info/#25) Plans for communicating important protocol modifications 7

(eg, changes to eligibility criteria, outcomes, analyses) to relevant parties (eg, investigators, REC / IRBs, trial participants, trial registries, journals, regulators)

Consent or assent [#26a](https://www.goodreports.org/reporting-checklists/spirit/info/#26a) Who will obtain informed consent or assent from potential 8

trial participants or authorised surrogates, and how (see Item 32)

Not Applicable,

No ancillary studies

Consent or assent: ancillary studies

[#26b](https://www.goodreports.org/reporting-checklists/spirit/info/#26b) Additional consent provisions for collection and use of

participant data and biological specimens in ancillary studies, if applicable

Confidentiality [#27](https://www.goodreports.org/reporting-checklists/spirit/info/#27) How personal information about potential and enrolled

participants will be collected, shared, and maintained in order to protect confidentiality before, during, and after the trial

6-8

Declaration of interests

[#28](https://www.goodreports.org/reporting-checklists/spirit/info/#28) Financial and other competing interests for principal 8

investigators for the overall trial and each study site

Data access [#29](https://www.goodreports.org/reporting-checklists/spirit/info/#29) Statement of who will have access to the final trial 8

dataset, and disclosure of contractual agreements that limit such access for investigators

Ancillary and post #30 Provisions, if any, for ancillary and post-trial care, and for

Not Applicable,

trial care compensation to those who suffer harm from trial participation

No ancillary studies, Inspection only

Dissemination policy: trial results

[#31a](https://www.goodreports.org/reporting-checklists/spirit/info/#31a) Plans for investigators and sponsor to communicate trial 8

results to participants, healthcare professionals, the public, and other relevant groups (eg, via publication, reporting in results databases, or other data sharing arrangements), including any publication restrictions

Dissemination policy: authorship

[#31b](https://www.goodreports.org/reporting-checklists/spirit/info/#31b) Authorship eligibility guidelines and any intended use of 9

professional writers

Dissemination policy: reproducible research

[#31c](https://www.goodreports.org/reporting-checklists/spirit/info/#31c) Plans, if any, for granting public access to the full 8

protocol, participant-level dataset, and statistical code

# Appendices

Informed consent materials

[#32](https://www.goodreports.org/reporting-checklists/spirit/info/#32) Model consent form and other related documentation given to participants and authorised surrogates

Additional

file 2

Not Applicable,

No ancillary studies

Biological specimens [#33](https://www.goodreports.org/reporting-checklists/spirit/info/#33) Plans for collection, laboratory evaluation, and storage of

biological specimens for genetic or molecular analysis in the current trial and for future use in ancillary studies, if applicable

Notes:

- 11a: 5，Figure.1
- 13: 4-5, Figure.2
