# Supplementary material for: The clinical value of mNGS of bronchoalveolar lavage fluid versus traditional microbiological tests for pathogen identification and prognosis of severe pneumonia (NT-BALF):study protocol for a prospective multi-center randomized clinical trial
Source: Trials. 2024 Apr 22;25:276. doi: 10.1186/s13063-024-08112-x (PMC11036641; doi:10.1186/s13063-024-08112-x)
Supplement: Supplementary file 2 — Additional file 2. Informed consent. [file 13063_2024_8112_MOESM2_ESM.docx]

**Additional file 2：Informed consent**

**Informed consent**

(Version number: V4.0 Version date: August 9,2023)

**Dear volunteer subjects or legal representatives:**

We invite you to participate in a multicenter prospective clinical trial of NT-BALF (the clinical value of metagenomic next-generation sequencing of bronchoalveolar lavage fluid versus traditional microbiological tests for pathogen identification and prognosis of severe pneumonia). The study will be conducted in the Peking Union Medical College Hospital, Beijing Chao-yang Hospital, Tianjin Medical University General Hospital, and The Second Hospital of Hebei Medical University.

Before you decide whether you want to participate in the study, it is important to understand why the study is being conducted and what it means to participate. Please read the following information carefully and discuss it with friends, family, or your personal physician if you need to. The study physician or research staff will also explain the study to you. You are invited to ask the study physician or study staff any questions you may have before making a decision and signing this informed consent form, and to request more information if needed.

Even if you wish to participate in the study, you may not be eligible to participate. If you are not eligible to participate in the study, the study physician or

study staff will discuss the reasons with you.

**Research Background**

Early pathogen identification and the inappropriate application of antibiotics for pneumonia at early stages is the major reasons for the increased morbidity and mortality rates in patients with severe pneumonia. Traditional microbiological tests include smear, culture, serology, and limited molecular panels. Bacterial/fungal cultures have a longer detection time (3-5 days) and a lower positive rate. Serological testing and PCR require a priori assumption about the presence of a particular virus. Previous studies have shown that metagenomic next-generation sequencing (mNGS) has a higher sensitivity and broader pathogen spectrum than traditional microbiological tests. Recently, the possibility of using mNGS to identify non-sterile site specimens in the respiratory tract has been discussed.

**Objectives**

In our study, patients who are willing to participate in this study and diagnosed with severe pneumonia are taken as research objects. The purpose of this study is to evaluate the effectiveness and consistency of bronchoalveolar lavage fluid (BALF) mNGS in diagnosing and treating pulmonary infections in comparison to traditional testing methods. Furthermore, the aim is to demonstrate that weather the combination of mNGS and traditional testing methods could decrease 28-day call-cause mortality. Your participation will make an important contribution to obtaining such evidence so that other patients can benefit from your contribution.

**Methods**

This is an observational study, and subjects will be divided into two groups: the study group (mNGS plus traditional microbiological tests) and the control group (traditional microbiological tests alone group). The grouping method is randomization (like a lottery), and neither you nor the researcher can choose in advance which group to participate in. This Study is only for observational purposes, and intervention in the clinical diagnosis and treatment of subjects is entirely decided by the clinicians (not the study physicians). A total of 192 patients with severe pneumonia will be recruited from four large tertiary hospitals in China.

**Research procedures**

Before commencing any research-related activities, you first need to sign this informed Consent form. During the screening period, the investigator will collect your personal information, previous diagnosis and treatment history, your combined medication, and arrange for you to undergo blood routine, urine routine, liver function, kidney function, chest CT and other examinations. The vital signs, ventilator parameters, anti-infective regimen and laboratory indicators will be recorded every day from the screening period. If you are eligible for admission, the investigator will begin the study by perfoming bronchoscopy and collecting your alveolar lavage fluid within 48 hours of enrollment and sending the fluid for mNGS and/or traditional microbiological testing.

The study will last for 28 days, and you will be followed up at 28 days of enrollment, as required by the protocol. During the follow-up, the investigator will ask you about your physical conditions. The research procedures will be arranged as follows:

V1. Screening period: Collect your personal basic information, perform routine examination and blood biochemical examination

V2. Sampling period: Performing bronchoscopy and collect your alveolar lavage fluid within 48 hours of entering the EICU, and inspect the alveolar lavage fluid for mNGS and/or traditional microbial testing

V3. Treatment period: The clinician (not the study physician) will adjust the anti-infective regimen based on your test results

V4. Follow-up period: Your physical condition will be followed up for 28 days

**When to end**

The study will last for 28 days and you will be treated according to clinical practice throughout and after the study. You could withdraw from the study at any time without any reason. If you decide to withdraw from this study, please inform your study physician in advance. In order to ensure your safety, you may be required to undergo relevant examinations, which is beneficial to protect your health. Please keep this informed consent form. During the course of the study, the study physician, study funders, regulatory authorities, and ethics committees may terminate the study.

**The benefit**

Your health may improve by participating in this study, but we cannot guarantee that you will. Your participation in this study may help physicians learn more about the impact of alveolar lavage fluid mNGS on clinical diagnosis and adjustment of anti-infection protocols of severe pneumonia. Other patients with the same or similar conditions may benefit from this information in the future.

**Risks and possible implications of the study**

This study is mainly carried out by means of bronchoscopy, which is safe and has no allergic reaction and is harmless to human body. You may feel slightly uncomfortable during the process. We will adjust the intensity according to the patient's tolerance. If you experience any discomfort during the bronchoscopy, please

call your study physician in time for consultation.

You will need to tell your family or close friends that you are participating in a clinical study and that they can take note of the events described above. If they have questions about your participation in the study, you can tell them how to contact your study physician.

The two diagnostic methods of this program are currently carried out in clinical practice, so participating in this program will not increase your risk. You do not need to visit the hospital on time during the study period, or do some additional tests, which will not take up your time or cause trouble or inconvenience.

**Alternative options**

You can choose

- - Do not participate in this study and continue your usual care.
  - Participate in other studies.

Consult with your doctor about your decision.

**Reward or compensation**

You will not receive any remuneration for participating in the study. In order to compensate you for the inconvenience that may be caused by your participation in this study, the study will pay the cost of relevant examinations performed during your participation in this study.

**Medical expenses compensation for injuries caused by the study**

If your health does suffer from study-related damage as a result of participating in the study, please notify the study doctor immediately and the study doctor will be responsible for taking appropriate treatment measures.

Even if you have signed this informed consent form, you still retain all your legal rights. If your rights and interests are violated, you can contact the The Institutional Review Board of Peking Union Medical College Hospital, Chinese Academy of Medical Sciences & Peking Union Medical College in Beijing, China. Tel: 010-69156874.

**Confidentiality**

Your medical records will be kept in the hospital and the investigators, research authorities, ethics committees will be allowed access to your medical records. Your personal identity will not be disclosed in any public report of the results of this study. We will make every effort to protect the privacy of your personal medical data within the scope permitted by law.

Personal and medical information about you will be kept confidential and kept in a safe and secure place. At any time, you may request access to your personal information (such as your name and address) and modify it if necessary.

By signing this informed consent form, you consent to the use of your personal and medical information for the purposes described above.

**Subject Consent Statement**

By signing below, you confirm that you have read and understood this informed consent form.

I declare:

I had plenty of time to ask questions related to this study and my questions were satisfactorily answered.

I understand that I am voluntarily participating in the study and that I can withdraw from the study at any time without penalty and without any loss of any benefits or medical services to which I am entitled.

My personal health information can be used and passed along as described above and added to the research database. My personal information, medical records, and pathological specimens may be used in future studies other than this study after approval by the ethics committee for the following purposes, including investigators or other companies and individuals working for or with investigators:

An investigational diagnostic technique with greater insight into safety and efficacy was developed.

Patients were studied for other therapies.

A better understanding of the diseases involved in research;And improving the

efficiency, design, and methodology of future clinical studies.

I agree that my personal physician will be informed that I am participating in this study and that they can provide health information about me to the study physician.

I understand that I will not lose any legitimate rights and interests by signing this informed consent form. I will receive a signed and dated copy of the informed consent form. By signing, I agree to participate in the study.

Signature of subjects: Date:

Name (in block letters): Subject Contact number: Signature of legal representative (if applicable): Date: Legal representative’ name (in block letters):

**Statement by the investigators**

I confirm that the details of the study, particularly the possible risks and benefits of participating in the study, were explained to the patient.

Investigator's signature: Date:

Investigator's name (in block letters): Investigator's Contact number:
